# Distribution of ferritin chains in canine lenses with and without age-related nuclear cataracts

**Published:** 2009-11-20

**Authors:** M. Goralska, S. Nagar, L.N. Fleisher, M.C. McGahan

**Affiliations:** Department of Molecular Biomedical Sciences, North Carolina State University, Raleigh, NC

## Abstract

**Purpose:**

It was determined in an earlier study that ferritin-heavy (H) and -light (L) chains in lens fiber cells are modified in comparison to those in lens epithelial cells. The purpose of the present study was to determine whether changes in ferritin chain characteristics are developmental, age-related, or associated with cataractogenesis, by analyzing the distribution of modified chains throughout the lens fiber mass.

**Methods:**

After removing the capsule, noncataractous and cataractous lenses were separated into six layers of fiber cells. The content of ferritin H and L chains in each layer was determined by western blotting with chain-specific antibodies. The level of ferritin complex (450 kDa protein made up of assembled L and H chains) was determined using the enzyme-linked immunosorbent assay. The ability of ferritin complex to bind iron was assessed by in vitro labeling with ^59^Fe.

**Results:**

Fiber cell ferritin L chains were 30 kDa (modified from the normal 19 kDa), and were present at the highest level in the outermost layers of both cataractous and non-cataractous lenses. The amount of modified L chains decreased gradually in the inner layers of the fiber mass, and was undetectable in the inner two layers of cataractous lenses. The ferritin H chains were also modified to 12 kDa (perhaps truncated from the normal 21 kDa size) in both cataractous and non-cataractous lenses. Similar levels of this modified H chain were found throughout the normal lens. Interestingly, in cataractous lenses, the modified H chains were found in decreasing amounts towards the interior of the lens, and were undetectable in the nucleus. However, in these cataractous lenses, the normal-sized ferritin H chains (21 kDA) appear in small quantities in the outer fiber layers, and increase in quantity and size (to 29 kDa) in the inner layers. This observation was best seen and demonstrated in advanced cataracts. Ferritin, which can bind iron, was found mainly in the outer layers of the lens fiber mass of normal lenses, but was more evenly distributed in fiber layers from cataractous lenses.

**Conclusions:**

Both ferritin H and L chains were modified in lens fiber cells from normal and cataractous canine lenses. These modifications were not age-related, and most likely occur during the differentiation of epithelial cells to fiber cells, since only normal-sized chains have been found in lens epithelial cells. In addition, there was a specific and distinct distribution of these modified chains throughout the lens fiber mass. The most striking differences between normal and cataractous lenses fiber cells were the appearance of normal-sized ferritin H chains and the relatively even distribution of iron binding capacity throughout the fiber mass of the cataractous lenses. These differences may reflect a response of the lens to increased oxidative stress during cataractogenesis.

## Introduction

Aging lenses develop a barrier preventing the flow of antioxidants from the metabolically active cortex to the lens nucleus [1]. As a result, the lens center, particularly an aging lens center, has low levels of reduced glutathione, and may be more susceptible to oxidative damage, which is considered to be a major factor in the development of age-related, nuclear cataracts [2]. Iron is implicated in cataract development, due to its ability to catalyze the formation of free radicals. Aging cataractous lenses have higher levels of iron [3] and an increased capacity for free-radical formation [4]. Excess iron is normally safely stored in ferritin, a ubiquitous protein with a highly conserved structure. Ferritin is a heteromultimeric complex consisting of 24 subunits of two types: light (L)-chain (19 kDa) and heavy (H)-chain (21 kDa). The L and H chains are assembled in a tissue-specific ratio. Each subunit has a complementary role in storing iron. The H chain has ferroxidase activity and facilitates iron oxidation and uptake into ferritin. The L chain translocates ferric ions into the core of ferritin for long-term storage [5]. Differences in total ferritin concentration, the structure of the chains, and their ratio can significantly alter the ability of ferritin to control redox-active, free iron, and to protect cells from iron-catalyzed oxidative damage [6].

Ferritin is present throughout the entire noncataractous and cataractous lens [3,7]. However, ferritin chains are significantly altered in lens fiber cells, in comparison to those of lens epithelial cells and other tissues [7]. Chain modifications identified in human and canine fiber cells include truncation (H chains; 10-12 kDa), increased size (canine L chains; 30 kDa), acidification, and polymorphism of both chains, which increases with age [7]. Chain modifications take place early, since altered chains are present in very young individuals, and accumulate with age in lens fiber cells [7]. It is likely that most of these significantly altered chains would not assemble into functional ferritin, and could remain as free chains. However, a small amount of ferritin of the proper size (450 kDa), capable of binding iron, has been detected in lens fiber cells by highly sensitive labeling with radioactive iron [7]. There are also differences in the characteristics of ferritin chains, between cataractous and transparent canine lenses of similar age. The predominant form of H chain present in cataractous lenses is a 29 kDa protein, which is larger than the 12 kDa identified in noncataractous lenses. This 29 kDa H chain can assemble into ferritin, which in cataractous lenses has a higher affinity for iron than ferritin from transparent lenses [8].

Analysis of the distribution of ferritin and ferritin chains in different layers would provide important information about the processing of these proteins and the roles they play in the lens. This includes how ferritin and ferritin chains are modified during differentiation from epithelial cells to fibers, during the process of aging, and, more specifically, whether these modifications are related to aging and/or cataractogenesis. Furthermore, these studies will help to determine if there are unmodified ferritin chains in specific layers of fiber cells that cannot be detected in protein-rich homogenates of whole lenses. A clearer understanding of the distribution of ferritin and ferritin chains in different lens layers would provide important information on the possible involvement of ferritin in the prevention of lens opacification caused by iron-induced oxidative damage.

## Methods

### Lens collection

Eyes were obtained postmortem from mixed-breed dogs euthanized at the Johnston County, North Carolina Animal Shelter. Five normal lenses were from 1- to 10-year-old dogs. Three cataractous lenses were from 8- to 10-year-old dogs. They were categorized as early-cataract (2 lenses) and advanced-cataract (1 lens), according to the severity of visible opacities.

### Fractionation of lenses

The lens capsule with epithelial cells was removed. The diameter of the total lens was measured and layers of fibers were peeled off using forceps under a dissecting microscope. The diameter of the remaining part of the lens was measured after each layer of fiber was removed. Each lens was dissected into six similar-size regions, and labeled in the order in which they were isolated (L1, the outmost L6, and the nucleus). The fibers of each lens region were collected in a small volume of phosphate buffered saline.

### Preparation of homogenates

Lens fiber fractions were sonicated in 10 mM Tris/HCl buffer, pH 7.4, containing 2% SDS and protease inhibitor cocktail for mammalian cells (Sigma, St. Louis, MO). The concentration of protein in the homogenates was determined using the BCA Protein Assay Kit (Pierce Biotechnology, Rockford IL).

### Immunodetection of ferritin chains in fiber homogenates

Samples containing 50 µg of protein were separated by 15% SDS-PAGE. Proteins were transferred to nitrocellulose membranes (Hydrobond ECL; Amersham Biosciences, Buckinghamshire England) by semidry blotting at 20 V for 20-30 min*.* Chain-specific antibodies and TrueBlot HRP-anti-rabbit IgG antibodies (eBioscience, San Diego, CA) were used for immunodetection of ferritin chains, as described previously [9]. We designed the peptides corresponding to H- and L-chain-specific amino acid sequences of canine ferritin. The antisera were produced in rabbits immunized with these peptides conjugated with Keyhole limpet hemocyanin by Research Genetics Inc. (Huntsville, AL). Immunoreactivity was determined using an ECL Western Blotting Analysis System (Amersham Biosciences). The images were digitized and evaluated with UN-SCAN-IT gel software (Silk Scientific, Orem, UT).

### In vitro ^59^Fe labeling of assembled ferritin

Samples containing 1-1.5 mg of protein were incubated with ^59^ Fe as ferric chloride (15.8 mCi/ml; 40.3m Ci/mg; PerkinElmer, Boston, MA) or as ferrous sulfate (1 mCi/ml; 42.24 mCi/mg; PerkinElmer) for 1 h at room temperature. Labeled proteins were separated by 8% SDS-PAGE under non-reducing conditions. Ferritin binding of ^59^Fe was measured in an Instant Imager (Packard-Canberra, Rockville, MD). There was no difference between labeling ferritin with radioactive ferrous or ferric iron.

### Measurement of ferritin content by enzyme-linked immunosorbent assay in canine lens fiber homogenates

Ferritin concentration was measured by a sandwich enzyme-linked immunosorbent assay (ELISA) using goat anti-horse ferritin and HRP-labeled goat anti-horse ferritin antibodies (Bethyl Laboratories Inc.), as described previously [10].

## Results

### Morphological characteristics of lenses and separation of lens fiber cells into layers

**Figure 1:**
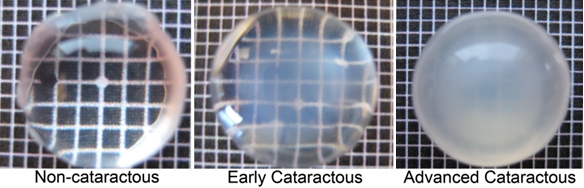
Photographs of representative noncataractous and cataractous canine lenses used in the experiment. Lenses were removed from eyes, placed on black-and-white grids to demonstrate the presence of opacities, and photographed.

The lenses with early cataracts showed central opacities and a transparent rim. The more advanced, mature cataractous lens was opaque throughout (Figure 1). The thickness of separated layers of fiber cells was measured, and the results were averaged within corresponding layers of all lenses, as shown in Table 1.

**Table 1:** The size of lens fiber layers L1-L6.

**Total lens diameter (mm)**	**Thickness of layers (mm)**					
	**L1**	**L2**	**L3**	**L4**	**L5**	**L6**
8.8±0.4	0.9±0.2	0.9±0.2	0.7±0.1	0.9±0.2	1.5±0.3	3.9±0.4

Values obtained for corresponding layers of all lenses were averaged and expressed in millimeters of thickness.

**Figure 2:**
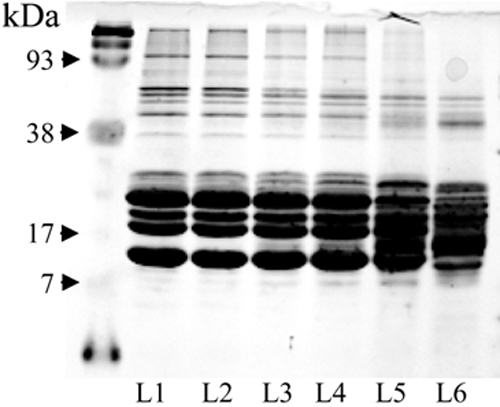
Total protein profile of different layers of fiber cells. A 50 μg sample (from a 10-year-old dog’s transparent lens) of protein homogenate from each layer was separated by 15% SDS PAGE under reducing conditions. Proteins were stained with coomassie blue.

The SDS-PAGE total protein profiles of homogenates of fiber layers were used to assess the separation of the lens into different regions. There were differences in the protein profiles of fiber cell lysates originating from different layers of lens. The differences included gradual elimination of higher molecular weight proteins (~ 90 kDa) from the lysates of layers 3-6 and visibly lower resolution of  lower weight proteins ( ~ 20 kDa) in layers 5 and 6 (Figure 2). The analysis of SDS-PAGE protein profiles confirmed that the dissection of each lens used in these studies was consistent.

### Characteristics and distribution of ferritin light and heavy chains throughout the noncataractous and cataractous lenses

**Figure 3:**
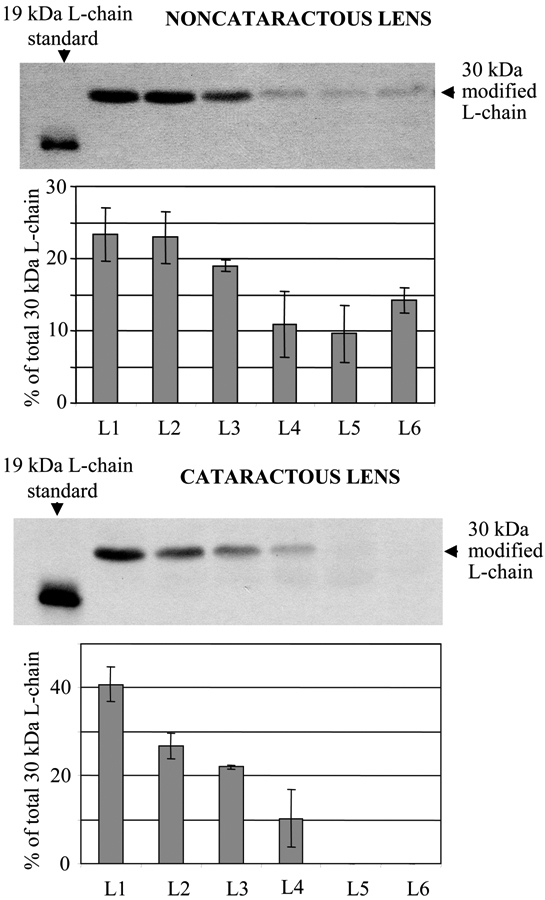
Western blot analysis of the distribution of 30 kDa ferritin L chains within the layers of fiber cells (L1-L6) of noncataractous and cataractous lenses. Fiber cell homogenates (50 µg proteins/sample) were separated by 15% SDS-PAGE under reducing conditions. Anti-canine ferritin L-chain antibodies were used to identify ferritin L chains. Canine liver ferritin was used as a standard. The representative blots show the L-chain distribution in lens fiber cells from a 1-year-old dog’s noncataractous lens and a 10-year-old dog’s lens with an early cataract.

In fiber cell homogenates of noncataractous and cataractous lenses, anti-ferritin L-chain antibodies detected a 30 kDa protein that was larger than the liver ferritin L-chain (19 kDa) used as a standard (Figure 3). These results were consistent with our previous findings that ferritin L chains present in canine lens fiber cells are modified, and that their concentration is lower in cataractous than in noncataractous lenses [7,8]. Both noncataractous and cataractous lenses showed similar distributions of L chains, which were located primarily in the lens cortex, with a large decline towards the nucleus. The nucleus of cataractous lenses did not have detectable amounts of the 30 kDa L chain (Figure 3). Since the distribution of modified ferritin L chains was the same in noncataractous lenses from young (1 year old) and old (8-10 years old) dogs, the results of the two groups were averaged. No normal-sized (19 kDa) ferritin L chains were detected in any layer of fiber cells from either noncataractous or cataractous lenses.

**Figure 4:**
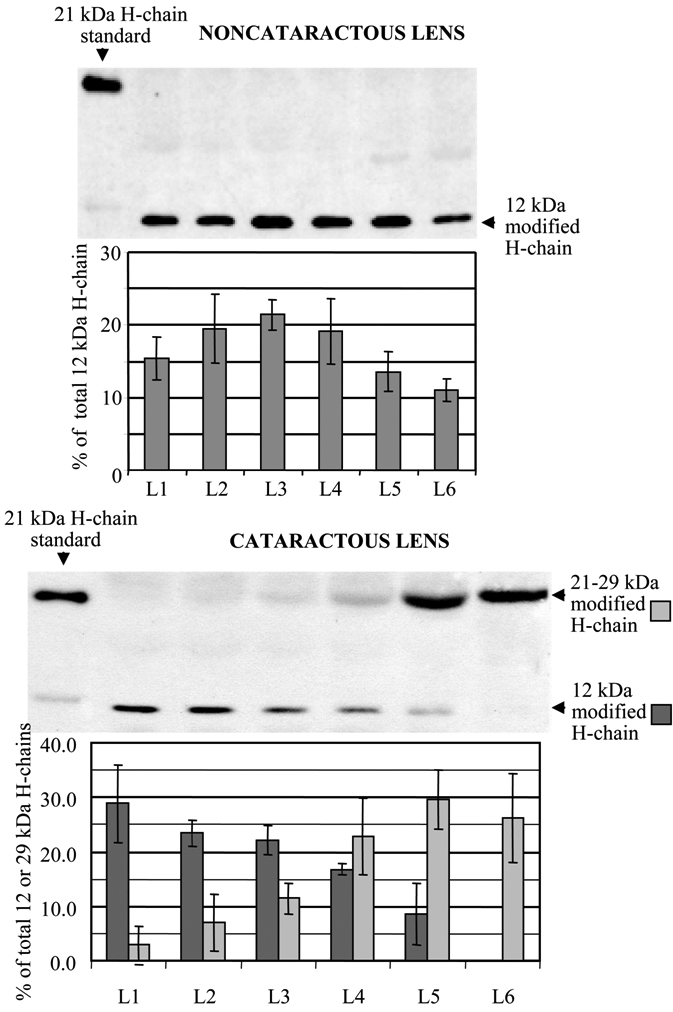
Western blot analysis of the distribution of ferritin H chains within the layers of fiber cells (L1-L6) from noncataractous and cataractous lenses. Fiber cell homogenates (50 µg protein/sample) were separated by 15% SDS-PAGE under reducing conditions. Anti-canine ferritin H-chain antibodies were used to identify ferritin H chains. Canine heart ferritin was used as a standard. The representative blots show the H-chain distribution in lens fiber cells from a 1-year-old dog’s noncataractous lens and a 10-year-old dog’s lens with an early cataract.

**Figure 5:**
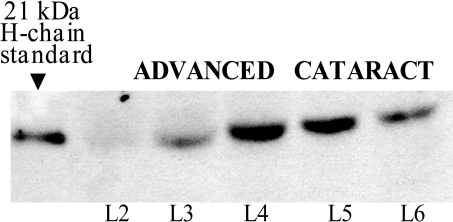
SDS-PAGE mobility of the 21-29 kDa ferritin H chains from different layers of an advanced cataractous lens. Fiber cell homogenates from the nucleus and inner cortex (50 µg/sample) were separated by 15% SDS-PAGE under reducing conditions. Anti-canine ferritin H-chain antibodies were used to identify ferritin H chains. Canine heart ferritin was used as a standard (21 kDa).

Antibodies against ferritin H chains detected a 12 kDa protein in fiber cells from noncataractous and cataractous lenses (Figure 4). This 12 kDa-modified H chain was present throughout the noncataractous lenses. Since the distribution of modified ferritin H chains was the same in noncataractous lenses from the young (1 year old) and the old (8-10 years old) dogs, the results were averaged together. In contrast, the distribution pattern of 12 kDa H chains in cataractous lenses was different. This chain was detected mainly in the outer layers of the lens, and its  content declined from the outer surface towards the nucleus. We have shown previously that cataractous lens fiber cells contain a differentially modified ferritin H chain (~29 kDa), as well as the 12 kDa H chain that was present in both lens types [8]. The 29 kDa H chain immunoreacted with anti-canine ferritin H-chain antibodies and was assembled into ferritin [8]. In the present study, using separated fiber cell layers rather than whole lens homogenates, western blot analysis revealed the polymorphic nature of this ferritin H chain. Surprisingly, the middle sections of cataractous lenses contained the 21 kDa H chain that is characteristic of other canine tissues. However, the molecular weight of the H chain increased to ~29 kDa towards the nucleus of the cataractous lens (Figure 4). The advanced cataract lens had a higher content of the 21-29 kDa chains, in comparison to lenses with early cataracts. Moreover, their presence extended to the fiber layers closer to the outer section of the lens, making the gradual mobility shift (from 21 to 29 kDa) of the chains through the cross-section of layers particularly well visible (Figure 5).

### Quantitation of ferritin in fiber cell layers from noncataractous and cataractous lenses

**Figure 6:**
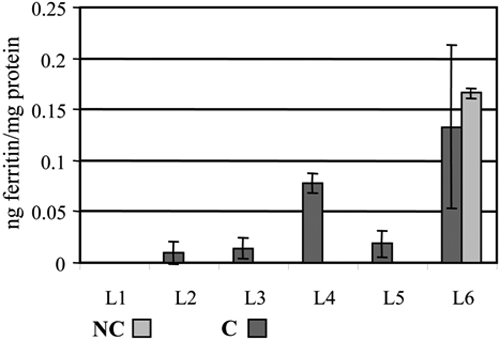
Levels of assembled ferritin in the layers of fiber cells from noncataractous (NC) and cataractous (C) lenses. Ferritin was measured by ELISA using polyclonal goat anti-horse ferritin antibodies, and expressed as ng ferritin/mg protein.

The distribution and content of ferritin throughout the lens sections were measured by ELISA using the antibodies that predominantly recognize the whole assembled ferritin molecule (polyclonal, goat anti-horse ferritin antibodies). They had a low affinity for unassembled free ferritin chains, as previously reported [7,8]. The results are shown in Figure 6. The distribution of ferritin did not follow that of ferritin chains, which were detected with chain-specific antibodies. Almost all ferritin detected by ELISA was found in the nucleus of noncataractous and cataractous lenses. Since there were no age-related differences in ferritin content and distribution in noncataractous lenses, the results were averaged.

### Iron-binding capacity of ferritin from the fiber layers of noncataractous and cataractous lenses

In our previous studies, we showed, by labeling with ^59^Fe, that lens fiber cell homogenates contain assembled ferritin (450 kDa) of a mobility equal to that of holoferritin from canine livers [7,8]. In order to determine the distribution of iron-binding ferritin within the lens, ferritin from the various fiber cell layers was labeled with iron in two valence states: ferric (^59^FeCl_3_) and ferrous (^59^Fe_2_SO_4_), which must undergo oxidation before incorporation into ferritin. There was no difference between in vitro labeling of ferritin with radioactive ferrous or ferric iron.

**Figure 7:**
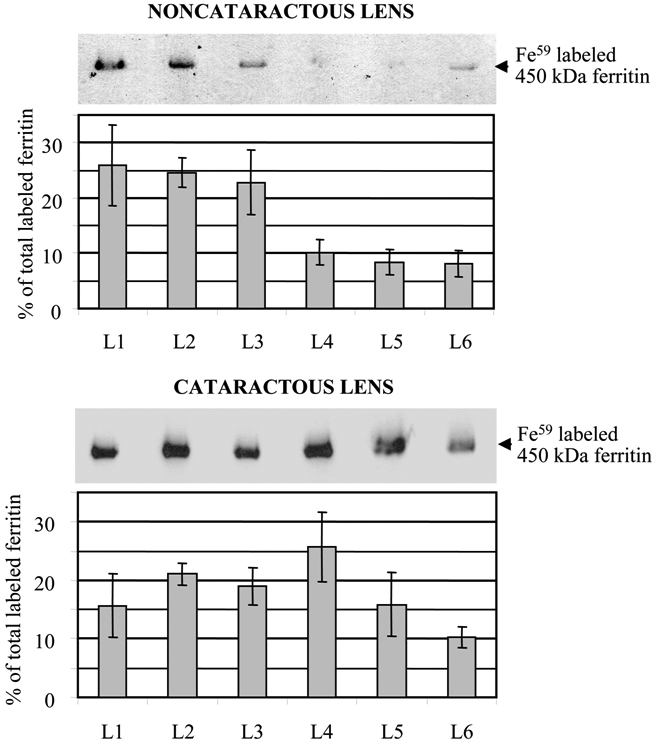
Distribution of ^59^Fe^-^labeled ferritin throughout different layers of lens fiber cells from noncataractous and cataractous lenses. Lens fiber cell homogenates containing 1500 µg of protein were incubated with ^59^FeCl_3_ and separated by 8% SDS-PAGE under nonreducing conditions. The mobility of the assembled ferritin complex was compared to that of 450 kDa canine liver ferritin standards. Incorporation of iron into each layer was expressed as a percent of incorporation into all layers combined. The autoradiograms shown are of a 1-year-old dog’s noncataractous lens and a 10-year-old dog’s lens with an early cataract.

Noncataractous lenses contained more iron-labeled ferritin in the three outer layers of fiber cells and significantly less in the inner regions of the lens (Figure 6). The distribution of iron-labeled ferritin in cataractous lenses was not significantly different between any of the layers (Figure 7).

This study also confirmed our previous findings that assembled ferritin from cataractous lenses binds more iron than does ferritin from noncataractous lenses [8].

## Discussion

Long-lived lens proteins undergo little turnover but show a high degree of enzymatic and non-enzymatic posttranslational modification. These modifications start early in life and continue as the lens ages, resulting in the accumulation of modified proteins [11]. Changes in lens protein structure lead to formation of high molecular weight aggregates with decreased solubility, a factor that contributes to the increase in the light-scattering characteristic of older lenses [12]. Lens proteins can also be modified by partial or complete proteolytic degradation, which is particularly dramatic during the transformation of epithelial cells into fiber cells [13]. Furthermore, proteosomal and non-proteosomal degradation systems are present in lens fiber cells. The activity of both systems declines with age, but is still detectable in mature fiber cells [14].

Analysis of the distribution pattern and characteristics of ferritin chains in lens fiber cells revealed similar changes to those described for lens crystallins, and consisted of size alteration, acidification, and polymorphism [7]. The modified 30-kDa ferritin L chains were present in fiber cells of canine lenses of all ages, including those with and without opacities [7,8]. The increase in size of the canine L chains, from 19 kDa (canine liver ferritin L-chain standard) to 30 kDa was tissue- and species-specific. Canine lens epithelial cells and human lens fiber cells contained the well-characterized 19 kDa L chain [7]. The modified 30 kDa L chain was only found in canine lens fiber cells, and its presence was not age-related or associated with cataractogenesis. Since the highest concentration of modified L chains was detected in the youngest fiber cells in the outermost layer of the lens fiber cells (Figure 3), its modification most likely occurs just after or during differentiation. The increased size of the ferritin L chain (by 11 kDa) may be due to interactions with specific intracellular compounds, such as fragments of lens crystallins. Recent reports indicate that low-molecular weight crystallin fragments are present in lenses of all ages and form covalent complexes with lens proteins [15,16]. The gradual decline of the 30-kDa L chain in older layers of fiber cells in the center of the lens may result from proteolysis or from additional conformational changes, which render the protein unrecognizable by anti-L-chain antibodies. Although the distribution pattern of L-chains was the same in all lenses examined, the overall content of this chain was lower in cataractous canine lenses, in comparison to noncataractous canine lenses [8].

Ferritin H chains identified in human and canine lens fiber cells, regardless of age, were smaller—and most likely truncated (10 kDa human, 12 kDa canine)—than those found in lens epithelial cells, which contain the well-characterized 21 kDa form [7]. This modification may occur during or just after differentiation of epithelial cells into fiber cells, since this chain is present in the outer layer of all lenses. This truncated H chain had a different distribution pattern in canine cataractous lenses, as compared to noncataractous lenses (Figure 4). In cataractous lenses, the distribution of the 12 kDa H chains followed the same pattern as that of the 30 kDa L chains. The content of the H chains was the highest in the outermost, younger fiber cell layer and gradually declined towards the center of the lens. Noncataractous lenses, regardless of age, exhibited similar levels of this modified H chain throughout all the lens layers. Interestingly, in the middle region of cataractous lenses, the appropriately sized 21 kDa H chain was detected, as well as the truncated, 12 kDa H chain (Figure 4). However, this 21 kDa H chain gradually increased in size in older lens fiber cells, and accumulated in the lens nucleus as a 29 kDa protein (Figure 5). It is likely that the gradual increase in size of the 21 kDa H chain occurred post-translationally and was unrelated to age, since noncataractous lenses of similar age did not have detectable levels of 21-29 kDa H chains. The formation and accumulation of 29 kDa H chains paralleled the cataract progression, and may reflect a causative relationship between the modification of ferritin H chains and cataractogenesis. Although we can only speculate on the mechanism by which truncated, 12 kDa H chains are replaced by 21-29 kDa H chains in older layers of fiber cells from cataractous lenses, accumulation of the normal-sized H chain may be required to limit iron-catalyzed oxidative damage in an environment with a limited supply of antioxidants [1]. Indeed, we have shown that in fiber cells from cataractous lenses, 21-29 kDa H chains assembled into a functional ferritin resulting in an increased H/L chain ratio [8]. Furthermore, the increased content of H-chain in assembled ferritin from cataractous lenses was associated with a higher iron-binding capacity, as compared to transparent lenses.

In order to determine whether the distribution of ferritin chains coincided with that of assembled ferritin, ferritin was measured throughout the lens by ELISA, using polyclonal anti-ferritin antibodies that preferentially recognized assembled ferritin but had a low affinity for ferritin L and H chains. In both cataractous and noncataractous lenses, almost all ferritin detected by ELISA was located in the lens nucleus (Figure 6). However, when ferritin was labeled with ^59^Fe, iron-binding ferritin was detected in all layers of fiber cells, although there was a trend to lower levels in the nuclear region (Figure 7). Such a contradiction can be explained by recognizing that labeling with ^59^Fe is a very sensitive method that permits detection of ferritin based on this protein’s ability to bind iron. In comparison, ELISA recognizes protein antigenic sites. The overall concentration of assembled ferritin in lens fiber cells, as measured by ELISA, is much lower than in lens epithelial cells (0.08-0.16 versus 60-200 ng/mg protein), and is close to the detection limit of this technique [7,10]. Therefore, ^59^Fe labeling may detect the presence of ferritin at a concentration below the ELISA’s detection level, but only if ferritin is functional—that is, capable of binding iron. Based upon these results, we hypothesized that the lens nucleus contains mainly improperly assembled ferritin, which retained its antigenity and therefore was measured by ELISA but has limited ability to bind iron. Indeed, ferritin associated with the water-insoluble fraction of lens protein homogenates has been reported in the nucleus of human lenses with age-related nuclear cataracts [3]. The abnormal ferritin detected in our study was found in the nucleus of all canine lenses, regardless of age or transparency. Therefore, it is unlikely that this abnormal ferritin contributed significantly to lens opacification; and since it had a very low affinity for iron, its contribution to lens anti-oxidant defense would be most likely limited.

We have previously shown that ferritin from cataractous lenses incorporates more iron than that from noncataractous lenses [8]. The present study confirmed previous results (Figure 7). The higher content of H-chain detected in ferritin from cataractous lenses [8] leads us to hypothesize that an increase in the H/L chain ratio may facilitate the uptake of iron by ferritin in cataractous lenses.

Based on our previous and current investigations, we hypothesized that L and H ferritin chains are modified during the differentiation of lens epithelial cells into fiber cells. As the lens ages, the chains undergo additional conformational changes, which renders them unrecognizable by anti-ferritin-chain antibodies, and/or degradable by proteolytic enzymes. The activity of proteolytic systems declines in mature fiber cells [14], which may contribute to the accumulation of altered, possibly aggregated, ferritin in the center of the lens. The age-related decrease in the chaperone activity of α-crystallins may also contribute to accumulation of improperly folded ferritin in the oldest fiber cells [17].

The distribution and chain content of the assembled ferritin reflect the need for lenticular tissue as protection against iron-catalyzed oxidative damage. Since both transparent and cataractous lenses contained iron-binding ferritin, the modified and normal-sized ferritin chains apparently assembled into functional ferritin complexes [8]. Lenses with age-related nuclear cataracts maintained a similar level of iron-binding ferritin throughout all lens layers (Figure 7). Furthermore, this ferritin had a higher content of assembled 21-29 kDa H chains, and therefore a higher affinity for iron [8]. Taken together, these changes in lenses with age-related nuclear cataracts would afford them better protection against iron-catalyzed oxidative damage.
